# Nano-plasmonic Bundt Optenna for broadband polarization-insensitive and enhanced infrared detection

**DOI:** 10.1038/s41598-019-48648-6

**Published:** 2019-08-21

**Authors:** Ehab Awad

**Affiliations:** Electrical Engineering Department, College of Engineering, King Saud University, Riyadh, 11421 Saudi Arabia

**Keywords:** Nanophotonics and plasmonics, Polaritons, Sub-wavelength optics, Mid-infrared photonics

## Abstract

Infrared detection devices are becoming miniature with micro or nano-scale size. The advantages of downsizing come on the expense of insufficient collection of infrared radiation. Therefore, utilizing nano-plasmonic optical antennas becomes mandatory. However, it is desirable to develop antennas with broad bandwidth, polarization insensitivity, wide field-of-view, and reasonable plasmonic losses in order to collect most of incident infrared radiation and enhance power absorption efficiency. Here, an innovative optical antenna (optenna) is proposed and demonstrated for the first time. It has a novel shape of Bundt baking-pan. The gold Bundt is arranged in a periodic array that can be placed on top of a thin-film infrared absorbing layer. The developed optenna can squeeze infrared electric and magnetic fields to 50 nm-wide area in order to enhance material absorption efficiency. It demonstrates polarization insensitivity and ultra-broad bandwidth with a large fractional-bandwidth within the near, shortwave, and midwave infrared bands. It shows a remarkable enhanced power absorption efficiency up to 8 orders of magnitude with a reasonable average power loss of −3 dB and 80° field-of-view. It can be promising for future applications in solar-cells, telecommunication photodetectors, shortwave cameras, and midwave microbolometers.

## Introduction

Infrared (IR) optical detection devices such as photodetectors, solar cells, cameras, and microbolometers are becoming smaller in size with a tiny effective active area in the range of few micrometers or even nanometers. Such small size has several advantages such as ultrafast optical response, low operating temperature, efficient cooling, efficient generation/ collection of photo-carriers, small pixel-size for high spatial resolution imaging, and the possibility of ultra-dense integration with other devices. However, that comes at the expense of the smaller aperture area of the device, and in turn inefficient collection of infrared energy. Therefore, the infrared plasmonic optical antennas come into play with its ability for efficient collection of optical energy from the large free-space area and concentrating it down to a device with a small size area^1–4^. The concentration of plasmonic field down to sub-wavelength nano-scale increases the effective absorption cross-section area of absorbing thin-film atoms, and in turn the materials absorption coefficient^2,4,5^.

Several optical plasmonic antennas have been reported in the literature so far. For example, and not as a limitation, some of it is mentioned here. A resonant half-wave dipole optical antenna for infrared (1.34 to 1.48 µm) concentration onto nanometer germanium photodiode is reported^6^. A broadband planar horn nano-antenna is demonstrated in the telecommunications wavelength range^7^. A compact dipole antenna can enhance coupling efficiency by 180 times^8^. A silicon-based plasmonic horn coupler with grooves can give a 27% coupling efficiency^9^. Extraordinary transmission through nano-array of coaxial ring apertures is demonstrated with almost four times enhancement in optical intensity^10^. A hybrid silicon-gold nano-particles antenna is realized and demonstrated^11^. A plasmonic spiral ring grating coupled to a vertical nano-optical antenna can enhance optical field intensity up to seven orders of magnitude^12^. A dielectric silicon nano-antenna is demonstrated with an ultra-low heating conversion^13^. A deep trench thin metal polarization-insensitive antenna is demonstrated^14^. A nano-antennas sandwiched between two graphene monolayers photo-detector were demonstrated^15^.

Not to mention that the optical antenna shape should be designed to collect optical energy from a large aperture free-space area and focus it down to a sub-wavelength nano-scale area. That requires careful design of the optical antenna shape in order to optical-impedance match between the free-space and detection device. In other words, it is required to minimize back-reflections of optical energy from the antenna. Such reflections can degrade the infrared energy collection efficiency^4^. Of course, the infrared energy excites surface plasmon polaritons (SPP) traveling waves on optical antenna metal surfaces, therefore it is better to have a relatively small antenna size to avoid SPP ohmic power losses on metal surfaces^16^. In addition, it is better to have a polarization-insensitive operation for the optical antenna, thus it can collect energy from any type of incident polarized or unpolarized infrared radiation. Moreover, it is better to have a broad bandwidth antenna to collect as much amount of energy contained within one particular wavelength band.

## Results

In this work, a novel optical-antenna (Optenna) is designed and numerically demonstrated for the first time. It has a shape of Bundt baking-pan. It has a concentric structure that is made of gold metal filled with air as a dielectric material. The conical concentric shape allows the optenna to easily impedance match between the free-space and an absorbing thin-film layer. It allows also to collect most of the incident infrared radiation due to its relatively large input aperture, then sub-wavelength nano-focus it beyond the diffraction-limit down to a 50 nm-wide area. In addition, it allows for polarization-insensitive operation because of its two-dimensional (2D) symmetry around its central axis. Moreover, it allows for a wide field-of-view (FOV) angle ≅ 80°. The 2D unit-cells array of Bundt optenna is tested using silicon-nitride (Si_3_N_4_) thin-film layer, as one example of the infrared absorption material, which is placed on top of a silicon substrate. The Bundt optenna dimensions are optimized for optical-impedance matching over three optical IR wavelength bands: near-infrared (NIR: 0.74–1 µm), shortwave infrared (SWIR: 1–3 µm), and midwave infrared (MWIR: 3–5 µm). Over each band, the designed Bundt optenna shows large optical enhancement of the optical field intensity as high as 12.4 dB (i.e. by ≅17.4 times), and dramatic enhancement of infrared absorption efficiency as high as ≅80 dB (i.e. 8 orders of magnitude) within the thin-film layer. The concentric horn structure of Bundt input stage allows for a broadband optical response, and a large fractional bandwidth as high as ≅42%. The non-resonant size of Bundt optenna is found to be relatively small when compared to operating wavelength, and thus its plasmonic ohmic-losses becomes reasonable. In addition, the small size makes the Bundt optenna more suitable for miniature IR detectors. Moreover, the choice of an air-filling dielectric instead of other dielectric materials reduces the plasmonic ohmic-losses on the gold surface as well, because the plasmonic field becomes less confined to the metal-dielectric interface^16^.

**Figure 1:**
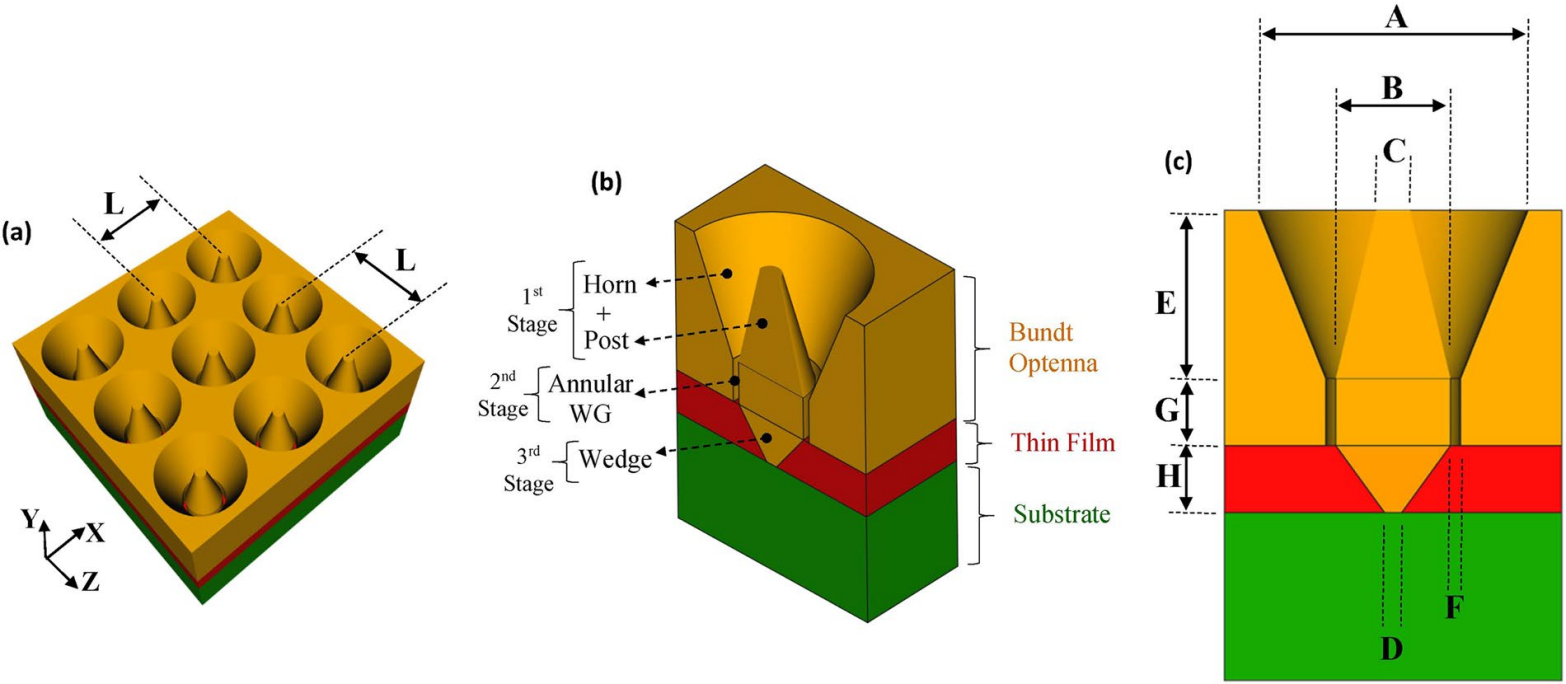
The schematic of the proposed infrared detection device with Bundt optenna. (**a**) A perspective view of 2D periodic Bundt array, (**b**) A vertical cross-section of unit-cell indicating different stages and the underneath semiconductor layers, (**c**) A vertical cross-section of unit-cell indicating different dimensions.

Figure 1 shows the schematic diagrams of a generally proposed infrared detection device with a Bundt optenna on-top of semiconductor thin-film detection layer and a thick bulk substrate. The optenna consists of a symmetric 2D periodic array of gold Bundt unit-cells, see Fig. 1a. As shown, the shape of the optenna unit-cell looks like a Bundt baking-pan. The separation between every two successive unit-cells is ‘L’. Figure 1b shows a vertical cross-section (X-Y plane) into one unit-cell together with its underneath layers. The Bundt consists of three stages. The first stage by itself consists of a coaxial (i.e. concentric) conical post and conical horn. The second stage is an annular straight waveguide (WG), and the third stage is a conical wedge. Figure 1c indicates different dimensions on the face of one unit-cell cross-section.

The incident free-space infrared radiation is almost totally collected by the Bundt array through the large aperture of the first stage (i.e. coaxial horns and posts). The coaxial horn and post with air-filling dielectric act like a metal-insulator-metal (MIM) structure with a conical flare. The Bundt first stage is end-fire excited by incident IR radiation to generate SPP on its gold surfaces, and result in a TE_11_ mode propagating along the MIM conical structure in the negative y-direction. Both the plasmonic electric and magnetic fields are squeezed gradually until they reach a sub-wavelength nano-wide annular area at the first stage output. This area equals π × [(B/2 + F)^2^ − (B/2)^2^] with a preset selected nano-width ‘F’ of 50 nm. The dimensions ‘A’, ‘B’, ‘C’, and ‘E’ of the first stage are optimized to best match optical-impedance between the coaxial horn and free-space on the Bundt input side. In other words to minimize the optical back-reflections and maximize the forward propagating transmitted signal.

In order to best match the optical-impedance between first stage output and third stage input (i.e. thin-film layer), an annular WG with annular width ‘F’ and air filling dielectric is placed between them as an intermediate second stage. The WG acts like a natural extension to the flared coaxial horn that propagates TE_11_ squeezed mode, and thus minimizes back-reflections. In addition, the annular WG length ‘G’ is optimized to maximize the transmission through the second stage into the thin-film layer. The finite length WG acts like a Fabry-Perot with multiple reflections between its input and output interfaces. The length ‘G’ is selected such that the round-trip total phase-shift of WG is 2 × G × k + Δφ = mπ, where ‘Δφ’ is the total excess phase-shifts due to reflections at WG input/ output interfaces, ‘k’ is the electromagnetic field propagation constant, and ‘m’ is an integer number^17^. This condition results in destructive interference among back-reflected waves at WG input, and thus constructive interference among transmitted waves at WG output.

The third stage of Bundt optenna consists mainly of a conical gold wedge. That is in addition to the Bundt base residing on the top of the thin-film layer. The wedge length ‘H’ is equal to the thin-film layer thickness. The conical wedge and Bundt base act together like a flared MIM structure that is excited by the SPP coming out of annular WG. This excited SPP mode propagates while spreading inside the thin-film absorbing layer. Therefore, the wedge allows excited SPP to penetrate deep inside the absorbing layer and thus increasing the overall absorption area inside the thin-film.

The silicon nitride (Si_3_N_4_) material layer was selected chosen as an example of absorbing thin-film with a thickness of H = 700 nm. While the substrate material is chosen to be a bulk silicon (Si). It is known that silicon nitride material has poor infrared absorption efficiency. Therefore, it is chosen here to illustrate the ability of Bundt optenna to significantly increase such weak IR absorption. And thus, it is expected to increase the absorption efficiency of other well-known good IR absorbing materials as well. The Bundt optenna array was tested over the NIR, SWIR, and MWIR wavelength bands. The dimensions of the Bundt stages are numerically optimized for best optical-impedance matching over most wavelengths of each band. In other words, to minimize reflections and maximize transmission while keeping almost flat optical-response across each band. It is found that there are four designs, each with specific dimensions, which can cover the three bands. Table 1 shows these designs with their corresponding dimensions. The following design rules were applied. For each wavelength range, the coaxial horn length ‘E’ should be ≥λ_max_ of that range. That is because at least one electric-field cycle should be existing along the horn length. Also, the inner perimeter of annular WG (i.e. π × B) should be ≥λ_max_ of the corresponding wavelength range. That is because the squeezed TE_11_ magnetic-field around annular WG perimeter cannot be smaller than λ_max_ for a mode to survive. In order to minimize the overall SPP ohmic loss on a gold surface, the ‘E’ and (π × B) are selected here approximately equal to λ_max_ (i.e. the minimum possible value). The dimension ‘A’ is selected to keep the coaxial horn symmetric between the inner and outer sides of annular WG. The dimensions ‘C’, ‘D’, ‘G’, and ‘L’ are selected through numerical iterations to minimize the Bundt back-reflections and maximize its overall transmission, i.e. it is optimized for best optical-impedance matching over each wavelength range. The largest Bundt unit-cell design is found to be for the MWIR band with size dimensions of 3.25 × 3.25 × 6.8 µm^3^. While the largest unit-cell aspect-ratio is found to be for NIR band with a value of 3.6:1. The dimensions of the four designs indicate an overall compact-size relative to the operating wavelength and a reasonable aspect ratio. It is worth mentioning that optenna designs ‘1’, ‘2’, ‘3’, and ‘4’ can be useful in applications such as solar cells, optical communications photodetectors, SWIR sensors/cameras, and MWIR thermal detection/imaging with microbolometers, respectively.

**Table 1:** The four designs of Bundt optenna that can cover the three IR-bands.

IR-band	Band range (µm)	Design	A	B	C	D	E	G	L
NIR	0.74–1	**1**	0.6	0.32	0.05	0.125	1	0.5	0.625
SWIR	1–2	**2**	1.4	0.7	0.05	0.25	2	1	1.425
	2–3	**3**	2	1	0.1	0.35	3	0.5	2.025
MWIR	3–5	**4**	3.2	1.6	0.2	0.55	5	1	3.25

The assigned dimensions’ are already indicated in Fig. 1, and they are measured in ‘µm’. The dimensions H = 0.7 µm and F = 50 nm are fixed for all designs.

**Figure 2:**
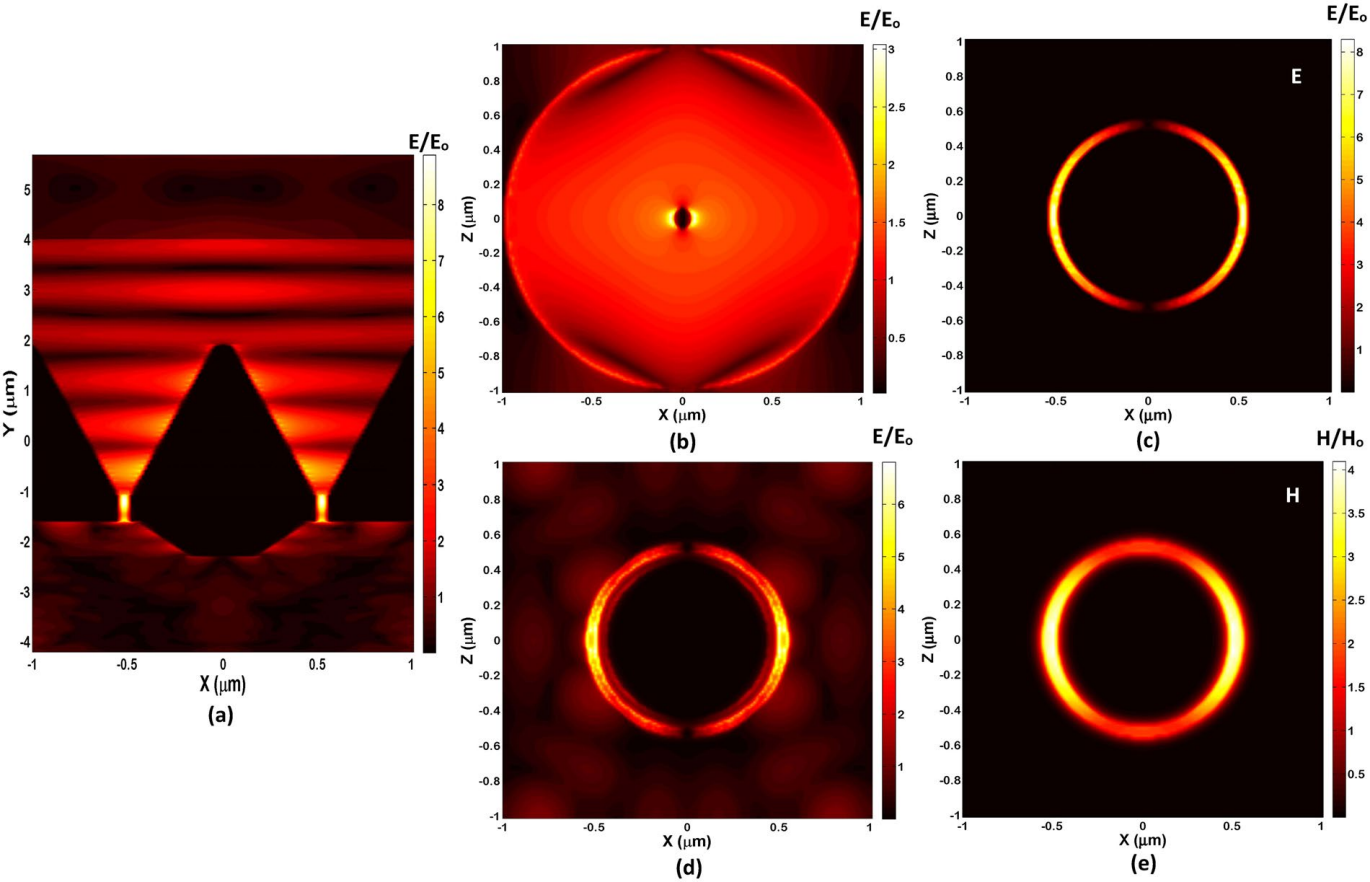
The 3D-FDTD simulations normalized fields within a unit-cell of infrared detection device at a wavelength of 2.3 µm, and TM incident polarization. The normalized electric-field magnitude (E/E_o_): (**a**) A vertical cross-section in a unit-cell, (**b**) A horizontal cross-section at the input of concentric horn, (**c**) A horizontal cross-section at the input of annular waveguide, (**d**) A horizontal cross-section just outside the annular WG (i.e. at entrance of the thin-film layer. The normalized magnetic-field magnitude (H/H_o_): (**e**) A horizontal cross-section at the input of annular waveguide.

The three-dimensional (3D) finite-difference time-domain (FDTD) simulations are performed on the gold Bundt optenna periodic array with underneath silicon-nitride thin-film layer and the silicon substrate. Figure 2 shows the normalized magnitudes of the electric (magnetic) field of one unit-cell when incident infrared light radiation propagates along the negative y-direction. The wavelength here is 2.3 µm with a TM linearly polarized wave along the x-direction. Figure 2a shows a vertical cross-section of a unit-cell (X-Y plane) at Z = 0 (similar to that in Fig. 1b,c). As shown, the SPP propagating electric-field inside the concentric horn are concentrated around the post because it has a small apex angle. The highest electric-field intensity is found to be around and within the 50 nm-wide annular WG. Within the thin-film layer, i.e. underneath Bundt, the high-intensity field propagates and spreads inside the layer with more SPP concentration around the wedge and Bundt base. This high-intensity field is absorbed and exponentially attenuated within the thin-film layer. It almost vanishes inside the underneath silicon substrate (extending beyond y ≤ −2 µm). The very small back-reflected electric-field from the structure appears alone at the top of the figure beyond the source. A Supplementary Video S1 is provided to illustrate the incident electric-field (magnitude) propagating through the Bundt optenna and the underneath absorbing layer at a wavelength of ≅2.3 µm.

Figure 2b–d show the electric-field of horizontal cross-sections (X-Z plane) at Bundt input, annular WG input, and WG output, respectively. While Fig. 2e shows the magnetic-filed of the horizontal cross-section at annular WG input. They illustrate the evolution of TE_11_ mode squeezing (compression) from free-space down to 50 nm-wide hot annular-area at the thin-film layer input. Figure 2b shows the normalized electric-filed of TE_11_ mode profile (TM-polarized) at the concentric horn input with more concentration around the center post. Figure 2c shows the squeezed electric-field magnitude of TE_11_ plasmonic mode at the annular WG input (i.e. concentric horn output). The normalized electric-field reaches a maximum of 8 at the annular WG ring, indicating an almost eight times improvement in the field strength (i.e. ≅18 dB), compared to the incident free-space radiation. Figure 2e shows the squeezed normalized magnetic-field magnitude for TE_11_ plasmonic mode at the annular WG input. It reaches a maximum of 4 at the annular WG ring, indicating an almost four times improvement in the field strength (i.e. ≅12 dB), compared to the incident free-space radiation. Note that the electric-field of TE_11_ mode vanishes at top and bottom of Fig. 2c, as the magnetic-field becomes totally directed along the y-axis with zero ‘x’ and ‘z’ components. Figure 2d shows the squeezed electric-field just outside the annular WG and inside the thin-film layer. The electric-field strength starts to attenuate due to optical absorption inside the thin-film.

## Discussion

The 3D-FDTD simulations are also utilized to characterize and evaluate the performance of Bundt optenna designs over its corresponding wavelength range.

**Figure 3:**
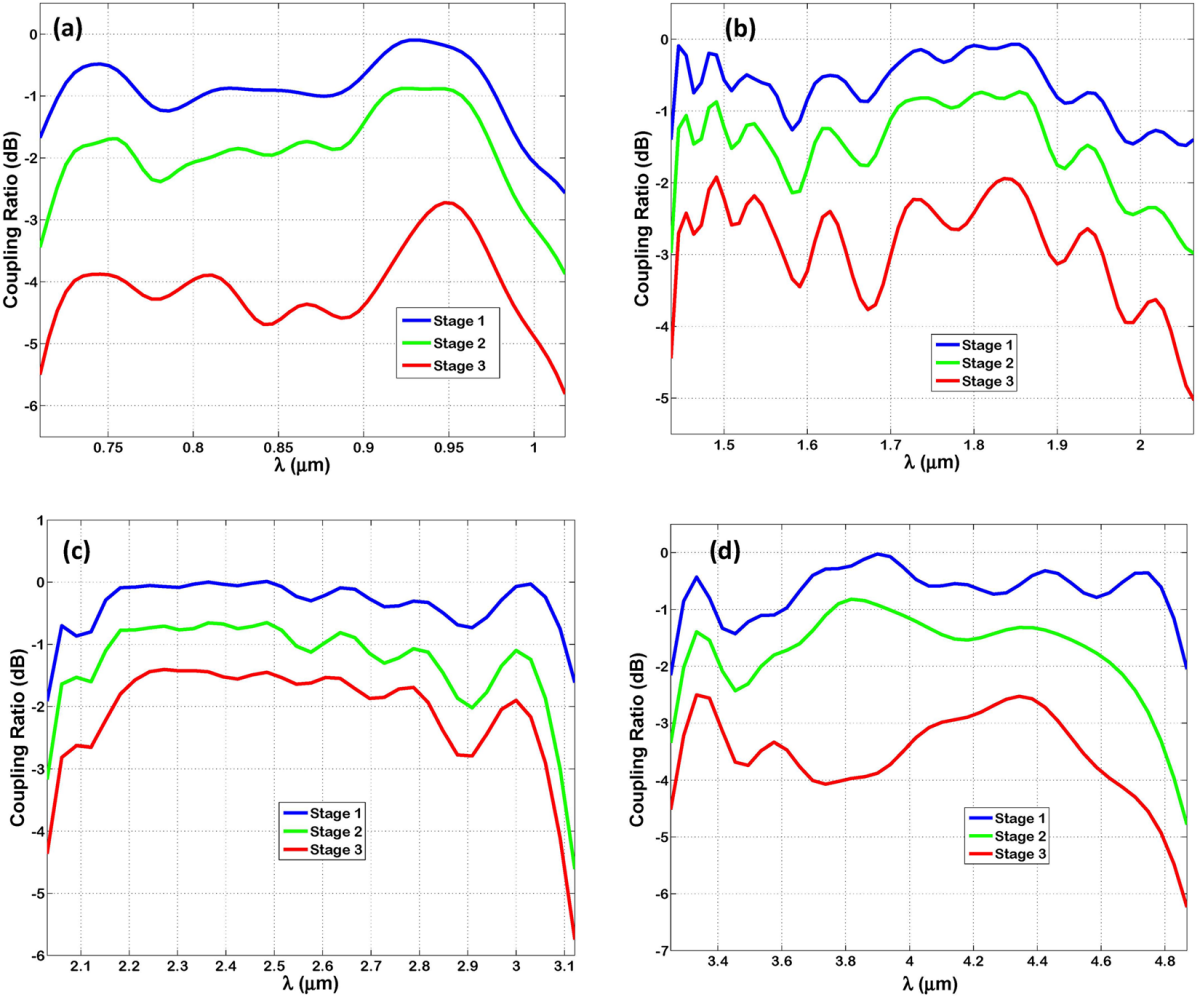
The coupling ratio estimated in decibels of TM-polarized infrared radiation to successive stages of Bundt optenna: (**a**) Design ‘1’, (**b**) Design ‘2’, (**c**) Design ‘3’, (**d**) Design ‘4’.

Figure 3 shows the coupling ratio (CR) in decibels of TM-polarized radiation into each Bundt optenna stage against the corresponding wavelength range. Figures (a), (b), (c) and (d) correspond to designs ‘1’, ‘2’, ‘3’, and ‘4’, respectively. The CR is defined as 10log_10_ (T), where ‘T’ is the power transmission measured at each stage input. For all designs, the CR into stage ‘1’ is considerably high, however it does not reach a value of zero-dB (i.e. T = 1) because of the inevitable little back-reflections (1-T) into free-space. The back-reflections are estimated to be ≤−7 dB over most of the tested bands. The CR within the third stage indicates a high collection efficiency of the optenna to incident free-space radiation. That is despite the relatively thick gold layer (E + G) of optenna on top of the absorption thin-film, which is supposed to obscure most of the incident light by back-reflections and metal ohmic losses. As expected, the CR of the second and third stages are less than that of the first stage due to SPP ohmic power loss on the gold surfaces of concentric horn and annular WG, respectively.

The 3 dB bandwidth (BW) of Bundt optenna is estimated from the CR curve of the third stage, as it indicates the amount of power transferred by optenna to the thin-film layer. The small ripples appearing on curves are due to the Fabry-Perot effect, which is due to the residual multiple reflections from different interfaces of optenna stages. Table 2 indicates the measured bandwidth for each Bundt design together with its estimated center-wavelength (λ_o_), and the calculated optical fractional bandwidth (O-FBW). The O-FBW is defined as the ratio of measured bandwidth to center-wavelength (i.e. BW/λ_o_). The table illustrates a broadband optical response (i.e. BW) with a considerably high optical fractional bandwidth ranging from ≅35% up to 42%.

**Table 2:** The wavelength range, bandwidth (BW), and center-wavelength (λ_o_) of different Bundt designs, measured in micrometer (µm). In addition to the percentages of optical fractional bandwidth (O-FBW).

IR-band	Design	Wavelength range	BW	λ_o_	O-FBW (%)
NIR	**1**	0.71–1.01	0.3	0.86	34.9
SWIR	**2**	1.43–2.06	0.63	1.75	36
	**3**	2.03–3.12	1.1	2.58	42.6
MWIR	**4**	3.25–4.86	1.61	4.06	39.7

**Figure 4:**
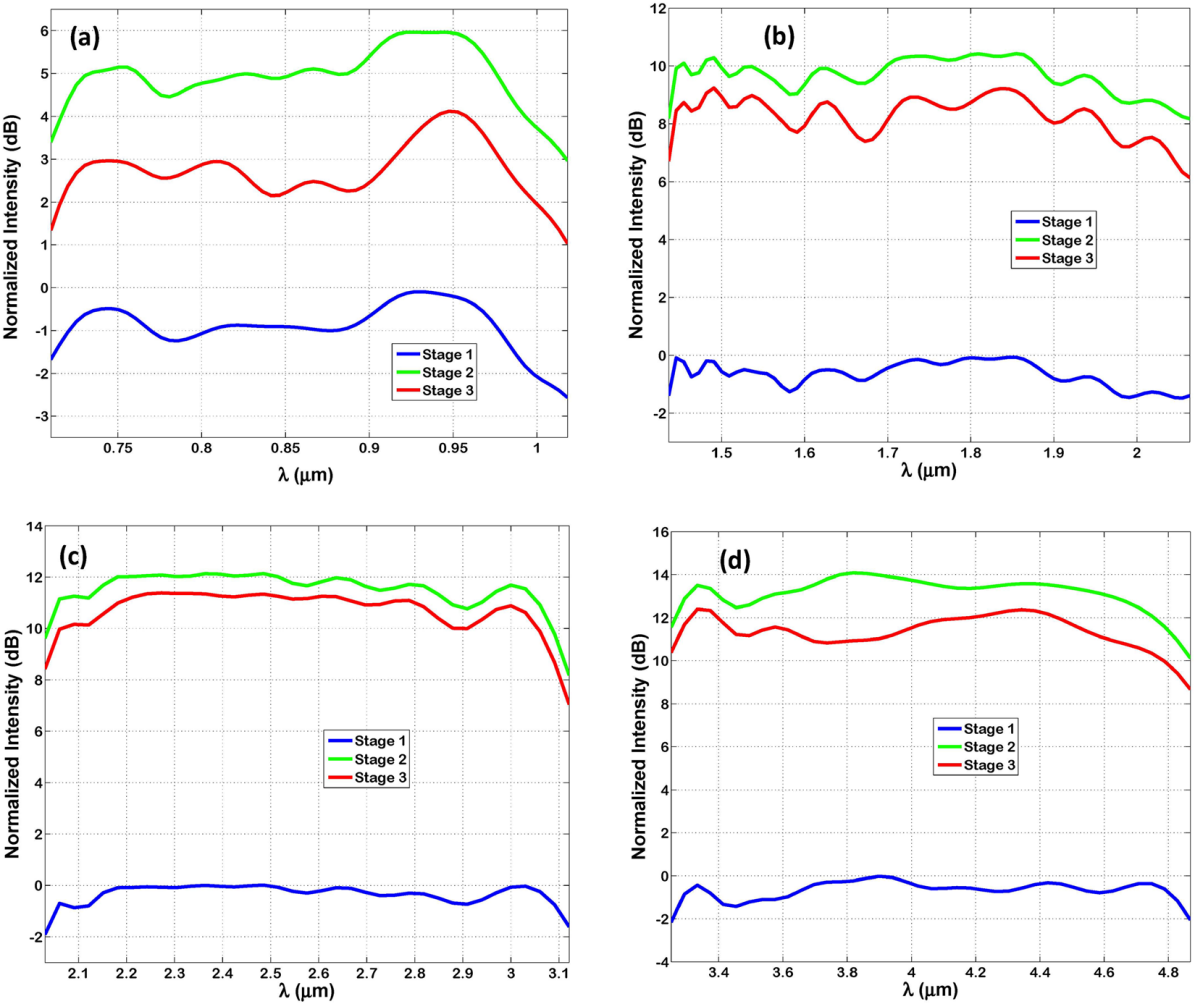
The normalized intensity measured in decibels of TM-polarized infrared radiation at each stage of Bundt optenna: (**a**) Design ‘1’, (**b**) Design ‘2’, (**c**) Design ‘3’, (**d**) Design ‘4’.

Figure 4 is similar to Fig. 3 except that it shows the normalized intensity at each stage. The normalized intensity is calculated as 10log_10_ (I/I_in_). Where ‘I’ is the intensity at each stage input, calculated as the transmitted power divided by the area. Whereas ‘I_in_’ is the total intensity at optenna input. Unlike Fig. 3, this figure shows improvement in normalized intensity while propagating from stage ‘1’ to ‘3’, despite ohmic power loss. This is because of fields squeezing and in turn dramatic reduction in its area. In other words, the gain in optical intensity becomes high despite power attenuation by SPP ohmic losses, because of the considerable compression of fields area. The importance of measuring intensity here is that it is the key parameter in controlling the materials absorption efficiency. That is because the effective absorption cross-section area of material atoms, and in turn the materials absorption coefficient is directly proportional to the incident optical field intensities (i.e. nano-focusing of fields), as mentioned earlier. Therefore, the absorption efficiency of the thin-film layer underneath the Bundt optenna is expected to increase, as illustrated later. However, note that the normalized intensity reduces from stage ‘2’ to ‘3’. That is because the area of the fields remains constant, and thus the SPP ohmic losses become dominant. The maximum achieved normalized intensity is 12.4 dB at the wavelength of 3.3 µm, which is almost a factor of ≅17.4 times, see Fig. 4d.

**Figure 5:**
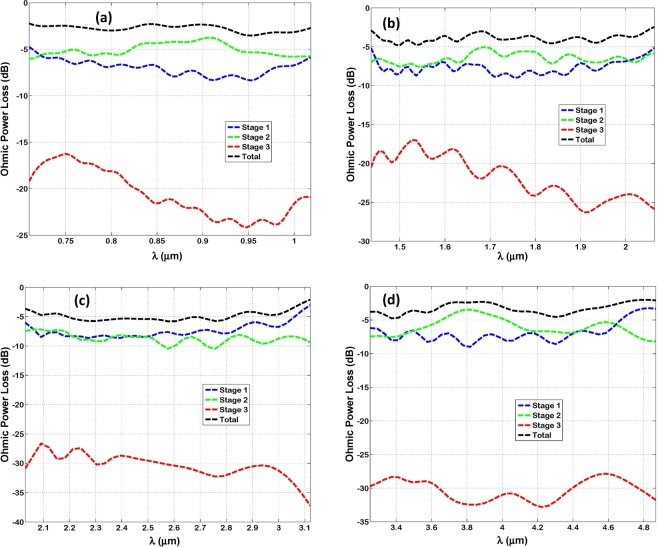
The SPP ohmic power losses measured in decibels of infrared radiation within each stage of Bundt optenna: (**a**) Design ‘1’, (**b**) Design ‘2’, (**c**) Design ‘3’, (**d**) Design ‘4’.

Figure 5 shows the ohmic power losses within each stage of optenna, in addition to its total power loss. The ohmic power loss is calculated as the difference between the net input and output power of each stage, divided by the Bundt total input power. The power loss inside the third stage is dissipated within the wedge and Bundt base, and it is the smallest one. However, the losses within stage ‘1’ and ‘2’ are higher than stage ‘3’. The loss of the relatively long concentric horn (stage 1) is often comparable or even less than that of the shorter annular waveguide (stage 3). That is because the annular waveguide has a very small width of 50 nm, thus it acts as a narrow MIM waveguide^16^ with expected losses higher than or comparable to that of the concentric horn. The summation of ohmic losses over all three stages (i.e. total loss) has a maximum, minimum, and an average value of ≅−2 dB, −6 dB, and −3 dB, respectively, for all designs and over different corresponding bandwidths. Which can be considered reasonable.

It is found that the polarization dependence of different designs over their corresponding bandwidths, is negligible (≅zero). The polarization dependence is defined as the ratio between the transferred powers by optenna in case of TM and TE incident polarizations. This polarization insensitivity is due to the 2D symmetry of Bundt structure around its central axis (i.e. along y-direction). Therefore, the Bundt optenna optical responses are always the same for all types of incident polarizations. That is advantageous as the optenna can collect most of the incident radiation power regardless of its polarization.

**Figure 6:**
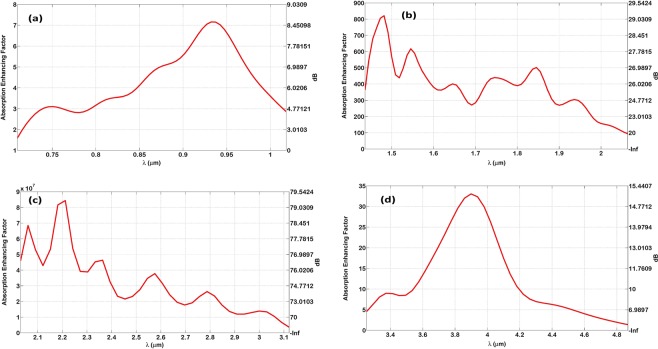
The absorption enhancement factor inside the thin-film layer due to Bundt optenna indicated in linear scale on the left axis, and in decibels on the right axis: (**a**) Design ‘1’, (**b**) Design ‘2’, (**c**) Design ‘3’, (**d**) Design ‘4’.

Figure 6 shows the absorption enhancement factor (EF) within the thin-film layer due to Bundt optenna array. The EF is defined as the ratio of absorbed power in the thin-film between the case with Bundt optenna and without Bundt optenna. The EF is shown in both linear scale and decibels on the left and right axis, respectively. The absorption enhancement factor is quite high and can reach a maximum value of ≅8.5, 29, 80, and 15 dB for designs ‘1’, ‘2’, ‘3’, and ‘4’, respectively. While the smallest possible EF is ≅2 dB for a couple of designs just at bandwidth edges. The very high EF of designs ‘2’ and ‘3’ are due to the very low extinction-coefficients of silicon nitride materials (i.e. small attenuation coefficients)^18^ within the corresponding wavelength bands. In other words, the silicon nitride absorption without optenna is almost negligible within these bands, while it becomes high and significant with Bundt optenna. Therefore, the power absorption enhancement becomes remarkable and significant due to optenna, especially within these two wavelength bands. For example, it can reach as high as ≅80 dB at the wavelength of ≅2.2 µm because the improved power absorption with optenna is divided by the negligible power absorption of the material without optenna. The ripples on some curves are because of the Fabry-Perot effect due to residual multiple reflections among optenna different stages. It is worth mentioning that the enhanced absorption efficiency of the thin film layer is due to the improved material absorption coefficient as the incident optical field intensities become much higher.

**Figure 7:**
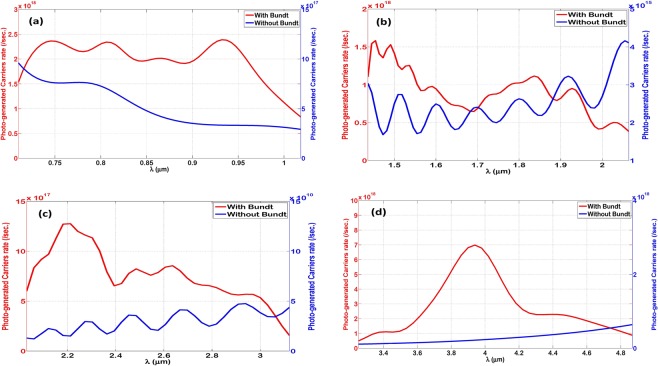
The photo-generated carriers rate (per second) due to optical absorption inside the thin-film layer in case with (red) and without (blue) Bundt optenna: (**a**) Design ‘1’, (**b**) Design ‘2’, (**c**) Design ‘3’, (**d**) Design ‘4’.

Figure 7 shows the estimated photo-generated carriers’ rate per second within the thin-film layer. It is calculated as a function of the wavelength by dividing the total amount of IR absorbed power inside the thin-film by the photon energy, assuming that each absorbed photon generates one electron-hole pair. The photon energy is calculated as hc/λ, where ‘h’ is the plank’s constant, ‘c’ is the light speed, and ‘λ’ is the wavelength. For assessment and comparison, the photo-generated carriers are estimated for both cases with and without Bundt optenna. Each case is plotted on a separate linear axis in order to illustrate the improvement in photo-generation rate. As shown, the improvement can be dramatic, especially for designs ‘2’ and ‘3’ as mentioned earlier. It is well-known that the detector quantum efficiency is directly proportional to the number of photo-generated carriers in addition to some other parameters depending on the IR detector type and specific structure. Therefore, the illustrated enhancement of generated carriers’ rate here can ascertain the improvement in the quantum efficiency of IR detection devices as well.

**Figure 8:**
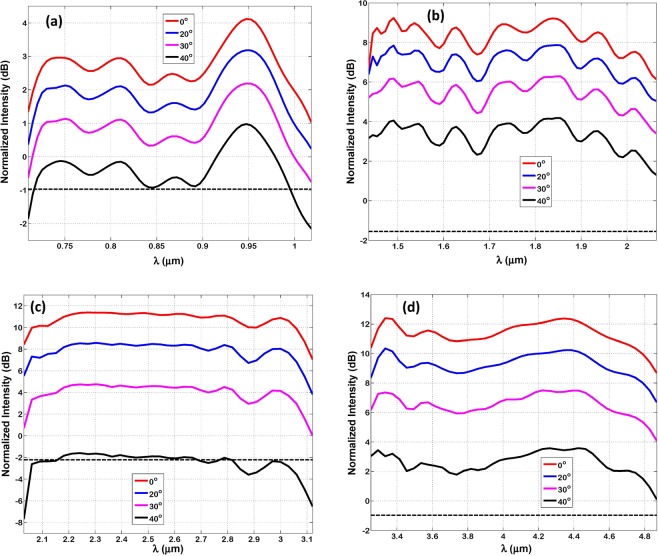
The Normalized intensity out of Bundt optenna as a function of incidence radiation angles: (**a**) Design ‘1’, (**b**) Design ‘2’, (**c**) Design ‘3’, (**d**) Design ‘4’. The dashed lines are for the case without Bundt optenna.

Figure 8 shows the normalized intensity out of Bundt optenna as a function of different incidence angle of free-space radiation. The incidence angle is varied beyond the normal incidence (i.e. zero angle) while monitoring the reduction in output normalized intensity. Of course, the incident radiation intensity is expected to reduce with angle as the incident fields’ components become smaller. The dashed lines correspond to the normalized intensity in case of thin-film without Bundt optenna. The dashed lines are considered here the limit of allowed intensity reduction, and thus the limit for maximum incidence angle. For all designs, the maximum incidence angle is found to be ≅40°, indicating a Bundt optenna field-of-view (FOV) ≅ 2 × 40° ≅ 80°.

Although the main focus of this work here is the numerical simulations of Bundt optenna, it is worth to give a hint on a suggested method for Bundt fabrication. The Bundt optenna may look complex in structure because of its three stages, however, it can be fabricated with simple steps. After deposition of the silicon nitride thin-film layer with a thickness ‘H’ on top of the thick silicon substrate, dry plasma etching can be used to pattern and etch conical holes array in the thin-film with a depth of ‘H’ and separations of ‘L’. Then, the gold can be deposited to fill up the holes and form wedges which constitutes the third stage of optenna. Afterward, a thick gold layer for both the first and second stages can be deposited on the entire absorbing thin-film layer with a total thickness of ‘E + G’. Then, the concentric conical horns and subsequently the annular waveguides can be formed inside this gold layer using focused ion beam milling, while controlling the milling area by changing the beam current and etching time.

In conclusion, a novel Bundt optical antenna array is developed and numerically demonstrated for the first time. The Bundt dimensions can be designed and optimized to cover the near, shortwave, and midwave infrared bands. The Bundt shows an ultra-broadband optical response with high fractional bandwidth of up to 42%. It can collect most of the free-space infrared radiation with a wide field-of-view of 80°. It can squeeze both the plasmonic electric and magnetic fields down to the 50 nm-wide area to enhance optical absorption efficiency within a thin-film detection layer. The gained intensity of squeezed fields can reach up to 12.4 dB with 18 dB and 12 dB enhancement in plasmonic electric and magnetic fields, respectively. Although the average ohmic power loss is −3 dB, the enhancement in power absorption reaches up to 8 orders of magnitude (i.e. 80 dB). The Bundt optenna is polarization insensitive and has a relatively compact size with a reasonable aspect ratio. The Bundt optenna can be promising for different nano-scale infrared detection devices such as solar cells, photodetectors, cameras, and microbolometers, with potential applications in energy harvesting, optical communications, imaging, sensors, and biomedical technology.

## Methods

The Bundt optenna device simulations were performed by three-dimensional finite-difference time-domain method using Lumerical solutions software package^19^. The device periodic structure is simulated using a unit-cell consisting of one Bundt optenna, thin-film absorbing layer, and substrate while applying periodic-Bloch boundary conditions on all unit-cell side-walls. The top and bottom boundary conditions of the unit-cell are set to perfectly-matched layers each with a max of 256 layers for stabilized simulation. The FDTD is performed using a non-uniform mesh setting with a maximum mesh step of 8 × 8 × 8 nm^3^. The simulation span in the y-direction is estimated to be ≅14 µm. The FDTD simulation time is set to 1000 femtoseconds. The mesh accuracy is set to 2 with the default conformal mesh refinement setting and minimum mesh step-size of 0.25 nm. Some convergence tests were conducted during the simulations to ensure reasonable numerical errors. The worst-case error was found to be ≅1% for the coupling ratio, which is considered small and acceptable.

A broad-band plane-wave source with linear polarization (TM or TE) is utilized. The source wavelength is set within each of the tested bands. The tested bands are the near-infrared (0.74–1 µm), shortwave infrared (1–3 µm), and midwave infrared (3–5 µm). Each wavelength-band is subdivided into 50 equally separated points, where the wavelengths can be swept between the band limits.

The gold and silicon material models are selected from the Lumerical default material library of Palik^20^ using complex refractive index constants (n, κ) at various operating wavelengths. Whereas the silicon nitride (Si_3_N_4_) material constants as a function of wavelength were obtained from the online thin-film database library^18^. A multi-coefficient model is used to fit the silicon-nitride material constants.

The total power absorption within the silicon nitride thin-film layer is calculated by integrating the power absorbed per unit volume (i.e. power density) over the absorbing layer. This is achieved by applying two volumetric monitors, one for the electric-field and the other for the refractive index within the absorbing layer. The absorbed power density is then calculated as 0.5 × ω × |E|^2^ × imag(ε)^19^. Where ‘ω’ is the angular frequency of absorbed light, ‘|E|’ is the electric-field magnitude, and ‘imag(ε)’ is the imaginary part of silicon nitride permittivity at the operating wavelength.

## Supplementary information

Suplementary video S1 - NanoPlasmonic Bundt optenna _ Ehab Awad
